# Using a Health Information Exchange to Characterize Changes in HIV Viral Load Suppression and Disparities During the COVID-19 Pandemic in New York City

**DOI:** 10.1093/ofid/ofad584

**Published:** 2023-11-29

**Authors:** Emma Tucker, Harry Reyes Nieva, Kayla Schiffer, Michael T Yin, Delivette Castor, Peter Gordon, Noémie Elhadad, Jason Zucker

**Affiliations:** Vagelos College of Physicians and Surgeons, Columbia University, New York, New York, USA; Department of Biomedical Informatics, Columbia University, New York, New York, USA; Department of Medicine, Harvard Medical School, Boston, Massachusetts, USA; Department of Biomedical Informatics, Columbia University, New York, New York, USA; Division of Infectious Diseases, Columbia University Irving Medical Center, New York, New York, USA; Division of Infectious Diseases, Columbia University Irving Medical Center, New York, New York, USA; Division of Infectious Diseases, Columbia University Irving Medical Center, New York, New York, USA; Department of Biomedical Informatics, Columbia University, New York, New York, USA; Department of Biomedical Informatics, Columbia University, New York, New York, USA; Division of Infectious Diseases, Columbia University Irving Medical Center, New York, New York, USA

**Keywords:** bioinformatics, COVID-19, disparities, health information exchange, HIV management

## Abstract

**Background:**

HIV viral suppression requires sustained engagement in care. The COVID-19 pandemic challenged care accessibility for many people living with HIV (PLWH). We used health information exchange data to evaluate the effect of pandemic-related disruptions in HIV care on viral load suppression (VLS) and to examine racial/ethnic disparities in VLS.

**Methods:**

We performed a retrospective observational cohort study of PLWH using data from a regional health information exchange in the New York City region between 1 January 2018 and 31 December 2022. We established 2 cohorts: PLWH who received HIV care in 2020 (cohort A) and PLWH who did not receive HIV care in 2020 (cohort B). We categorized HIV VLS outcomes as suppressed or not suppressed and calculated the prevalence of VLS between 2018 and 2022. We compared proportions using chi-square tests and used unadjusted and adjusted logistic regression to estimate the association among variables, including race/ethnicity, cohort, and VLS.

**Results:**

Of 5 301 578 patients, 34 611 met our inclusion criteria for PLWH, 11 653 for cohort A, and 3141 for cohort B. In 2019, cohort B had a lower prevalence of VLS than cohort A (86% vs 89%, *P* < .001). Between 2019 and 2021, VLS dropped significantly among cohort B (86% to 81%, *P* < .001) while staying constant in cohort A (89% to 89%, *P* = .62). By 2022, members of cohort B were less likely than cohort A to be receiving HIV care in New York City (74% vs 88%, *P* < .001). Within both cohorts, Black and Hispanic patients had lower odds of VLS than White patients.

**Conclusions:**

In New York City, VLS remained high among PLWH who continued to receive care in 2020 and dropped among PLWH who did not receive care. VLS was lower among Black and Hispanic patients even after controlling for receipt of care.

## Teaser

Health information exchanges offer the opportunity to study HIV trends across fragmented health systems. Here, we use health information exchange data to describe the impact of the COVID-19 pandemic on HIV viral suppression and to illuminate racial disparities in HIV outcomes.

The New York State End the Epidemic initiative lays out a plan to end the HIV epidemic. One pillar of the plan involves sustainably engaging people living with HIV (PLWH) in care to maximize viral load suppression (VLS). Achieving and sustaining VLS is crucial for improving individual health outcomes and preventing HIV transmission in the community [1]. Helping PLWH achieve undetectable HIV levels is especially critical, as many new infections are transmitted by individuals who know their diagnosis but are not virally suppressed [2].

Retention in HIV care is associated with increased HIV VLS, higher CD4 counts, and reduced overall mortality [3–5]. Nonetheless, significant racial and ethnic disparities exist across the HIV care continuum. Black and Hispanic PLWH are less likely than White PLWH to be linked to and retained in HIV care [5–8]. These delays and interruptions in HIV care exacerbate existing disparities in viral suppression and HIV-related mortality [9, 10].

The impact of COVID-19 on disruptions in care for PLWH is only starting to become clear. The Centers for Disease Control and Prevention recently released data showing a 32% reduction in HIV testing between the first and second quarters of 2020 [11]. Despite the expanded use of telehealth during the pandemic, several studies showed decreased retention in HIV care among PLWH [12–14], and preexisting racial disparities in HIV treatment may have worsened [15]. Data on HIV VLS during the COVID-19 pandemic are scarce, and current evidence shows mixed results depending in part on the populations studied [12–14, 16, 17]. A recent Veterans Affairs study found decreased rates of viral load (VL) testing during 2020 but no change in viral suppression among those PLWH who did receive VL testing [17].

New York City (NYC) has an estimated 87 500 PLWH and was hard-hit during the early stages of the COVID-19 pandemic [18, 19]. Access to sexual health services decreased significantly in 2020; nearly half of surveyed New York State–based sexual health clinics stopped offering follow-up HIV primary care between March and April 2020 [20]. Given the large number of medical facilities in NYC, fragmented care often presents a unique data collection challenge, as an individual patient may routinely receive care across multiple facilities and health systems [21]. Moreover, while the NYC Department of Health and Mental Hygiene (DOHMH) collects HIV diagnosis and testing data from across the city, its annual report does not provide information on month-to-month trends in VLS, quantity of VL tests performed per person, or associations between receipt of HIV care and VLS. Accordingly, our understanding of VLS among PLWH in NYC during the COVID-19 pandemic remains limited [22].

Health information exchanges (HIEs)—organizations that aggregate electronic health record (EHR) data across multiple health care systems and laboratories within a given geographic region—present a promising solution to better understand HIV outcomes with public health significance for PLWH. HIEs offer detailed patient-level information at a level of granularity and comprehensiveness neither typically available to local health departments nor possible with single-center studies.

Using HIE data, we examined receipt of HIV care, race/ethnicity, and VLS among PLWH in the NYC region before and after the onset of the COVID-19 pandemic (2019–2022). In particular, we aimed to (1) determine whether the prevalence of viral suppression differed between PLWH who continued to receive HIV care in 2020 and those who did not, (2) characterize racial and ethnic disparities in HIV viral suppression during the COVID-19 pandemic, and (3) demonstrate the utility of an HIE for studying HIV in a setting with significant care fragmentation.

## METHODS

### Study Design and Setting

We conducted a retrospective observational study of PLWH in the NYC region between 1 January 2018 and 31 December 2022 using deidentified data from Healthix: an HIE founded in 2007 that encompasses >20 million patients and 8000 health care facilities in the greater NYC region [23]. This study was approved by the institutional review board of the Columbia University Irving Medical Center (protocol AAAT1774), and reporting follows the STROBE guidelines (Strengthening the Reporting of Observational Studies in Epidemiology).

### Data

Our study data set was based on an extract from Healthix containing information on 5 301 578 patients with any of the following sexually transmitted infection tests performed between 1 January 2018 and 31 December 2022: chlamydia, gonorrhea, syphilis, hepatitis C, and HIV. Healthix uses a probabilistic matching system to assign individuals unique identifiers, allowing for tracking across sites [24]. The Healthix data set contained laboratory testing data, including diagnosis codes and laboratory tests, as well as demographic information such as gender, race, ethnicity, and zip code. As birth year data were not available, patient age was defined by the first age recorded in 2019.

For each patient in this extract, we obtained all available HIV screening and monitoring laboratory tests, which comprised 1543 LOINC (Logical Observation Identifiers Names and Code) and plain text description combinations (413 corresponding to HIV VL tests). In total, the file contained 901 266 VL laboratory tests from 228 facilities representing 77 360 unique patients.

### Participants

We defined PLWH as individuals with an *ICD* and/or SNOMED diagnosis code corresponding to HIV infection and ≥2 HIV VL tests over ≥2 years. The 2-part definition was largely adopted due to the common misuse of HIV diagnostic codes by some providers in reference to HIV screening tests and counseling, which may not represent true HIV infections. Any PLWH with inconsistent demographic information were excluded (ie, ≥1 individuals sharing a single patient identification; ∼0.1% of patient identifications).

### Cohort Assignment

**Figure 1.**
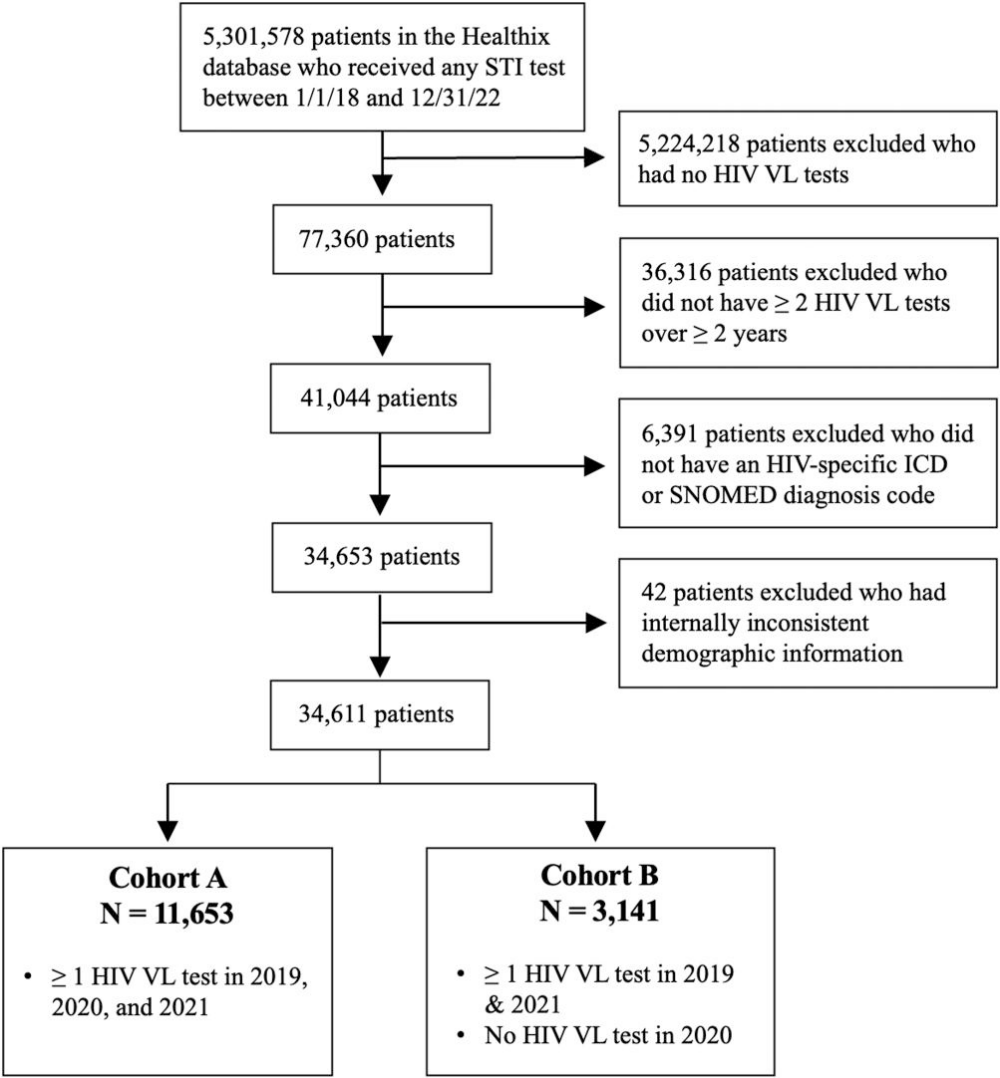
Cohort selection process. From an extract of 5 301 578 individuals with any sexually transmitted infection (STI) testing between January 2018 and December 2022, we identified 34 611 individuals who met our definition of people living with HIV (≥2 viral load [VL] tests over ≥2 years plus an *ICD* or SNOMED code corresponding to HIV). Of these, 11 653 had VL testing in 2019, 2020, and 2021 (cohort A), and 3141 had VL testing in 2019 and 2021 but not 2020 (cohort B). All data are drawn from Healthix, a large regional health information exchange based in New York City.

We established 2 cohorts (Figure 1). Cohort A consisted of PLWH who received HIV care throughout the pandemic, defined as patients who had ≥1 HIV VL tests during 2019, 2020, and 2021 (n = 11 653). Cohort B consisted of PLWH who did not receive care early in the COVID-19 pandemic, defined as having ≥1 VL tests in 2019 and 2021 but none in 2020 (n = 3141). This is in accordance with the NYC DOHMH, which defines “received care” as having at least 1 HIV VL or CD4 count/percentage in a given year.

### Outcomes and Covariates

#### HIV Viral Load Suppression.

HIV VLS was defined as a VL ≤200 copies/mL or ≤2.3 log_10_ copies/mL, consistent with the NYC DOHMH definition. Any value above this was classified as *unsuppressed*. We kept 1 VL result per PLWH per day. Patients with multiple discordant results from a single day (ie, 1 VL result ≤200 copies/mL and 1 result >200 copies/mL) were classified as *discordant* (<0.1% of observations). Discordant results were excluded from prevalence and odds ratio (OR) calculations. Results that were reported only as *detected* (<0.1% of observations) without a corresponding numeric value were also excluded from such calculations, as these results could represent values above or below 200 copies/mL.

#### VLS Across Time.

We selected each patient's last VL result from 2019 to represent viral suppression immediately prior to the onset of the COVID-19 pandemic (time point 1). Using the last VL value for a given year to approximate viral control is consistent with NYC DOHMH methodology. We then selected each patient's first VL result from 2021 (time point 2) to represent either initial receipt of care following loss to follow-up in 2020 (cohort B) or continued receipt of care (cohort A). Finally, we selected the last VL of 2022 (time point 3), which represents the most recent data that we have available (Figure 3).

**Figure 2.**
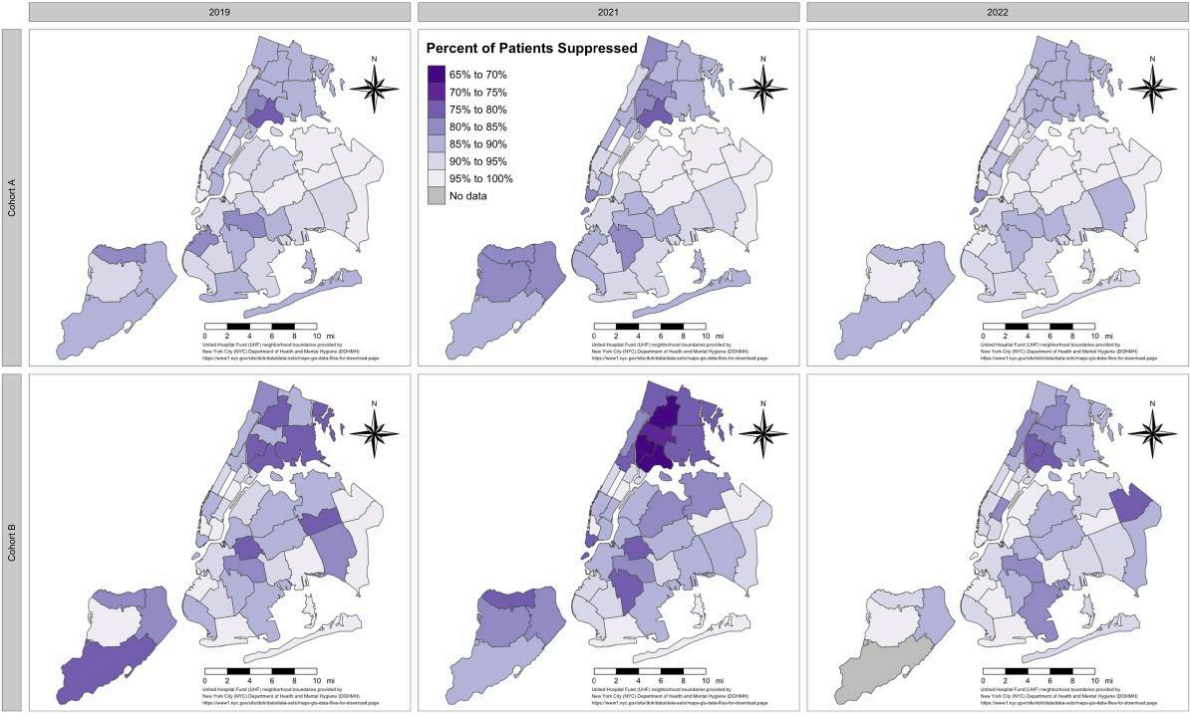
Prevalence of HIV viral load suppression (≤200 copies/mL) among people living with HIV in New York City by United Hospital Fund neighborhood boundaries across time (x-axis) and by cohort (y-axis). All data are drawn from Healthix, a large regional health information exchange.

To visualize the geographic disparities in viral suppression across NYC, we cross-referenced patient residential zip codes with United Hospital Fund neighborhood boundaries using shapefiles provided by the NYC DOHMH [25]; we then generated choropleth maps to illustrate the prevalence of viral suppression within each United Hospital Fund neighborhood across time and within both cohorts (Figure 2).

#### Race and Ethnicity.

As race and ethnicity were often conflated in EHRs, we combined values from both fields to create a composite variable wherein patients of Hispanic or Latino origin were classified as such and all other patients were categorized by race. For the purposes of calculating VLS prevalence estimates and constructing logistic regressions, patients were classified as *other* if they were initially classified as Asian, Native American or Alaska Native, Native Hawaiian or Pacific Islander, other, and more than 1 race, due to low cell frequencies (Table 1). Patients whose race/ethnicity was recorded as *declined* or *unknown* were excluded from these calculations.

**Table 1.** Baseline Demographic Characteristics of Cohorts A and B

	Cohort A^a^		Cohort B^b^	
	No.	% (95% CI)	No.	% (95% CI)
Overall	11 653	…	3141	…
Gender (*P* = .31)				
Female	3200	27.5 (26.7–28.3)	902	28.7 (27.1–30.3)
Male	8318	71.4 (70.6–72.2)	2199	70.0 (68.4–71.6)
Other, unknown, or declined	135	1.2 (1.0–1.4)	40	1.3 (.9–1.7)
Race (*P* < .001)				
Asian	211	1.8 (1.6–2.1)	40	1.3 (.9–1.7)
Black or African American	3344	28.7 (27.9–29.5)	1050	33.4 (31.8–35.1)
Hispanic or Latino	2572	22.1 (21.3–22.8)	580	18.5 (17.1–19.8)
Native American or Alaska Native	35	0.3 (.2–.4)	12	0.4 (.2–.6)
Native Hawaiian or Pacific Islander	11	0.1 (.0–.2)	3	0.1 (.0–.2)
White	1732	14.9 (14.2–15.5)	495	15.8 (14.5–17.0)
>1 race	3	0.0 (.0–.1)	1	0.0 (.0–.1)
Other race	1462	12.5 (11.9–13.1)	336	10.7 (9.6–11.8)
Unknown/declined	2283	19.6 (18.9–20.3)	624	19.9 (18.5–21.3)
Borough of residence (*P* < .001)				
Bronx	3094	26.6 (25.7–27.4)	914	29.1 (27.5–30.7)
Brooklyn	1941	16.7 (16.0–17.3)	625	20.0 (18.5–21.3)
Manhattan	3264	28.0 (27.2–28.8)	883	28.1 (26.5–29.7)
Queens	1868	16.0 (15.4–16.7)	368	11.7 (10.6–12.8)
Staten Island	200	1.7 (1.5–2.0)	79	2.5 (2.0–3.1)
Non–New York City	1225	10.5 (10.0–11.1)	251	8.0 (7.0–8.9)
Unknown	61	0.5 (.4–.7)	21	0.7 (.4–1.0)
Age, y, median (IQR)	51 (38–58)		51 (38–58)	

All patient data are drawn from Healthix, a large regional health information exchange based in New York City. *P* values are based on chi-square.

^a^People living with HIV who received HIV care in 2020.

^b^People living with HIV who did not receive HIV care in 2020.

#### Prepandemic Receipt of HIV Care.

To explore whether baseline receipt of HIV care differed by cohort prior to the COVID-19 pandemic, we compared the average number of annual VL tests per individual in 2019 between cohorts A and B.

#### Current Receipt of Care.

We considered patients to be currently in receipt of care if they had any VL test during 2022.

#### Role of COVID-19.

To determine whether more PLWH were lost to follow-up in 2020 as compared with the surrounding years, we calculated the percentage of patients with VL testing in 2019 and 2021 but not 2020, relative to those with testing in all 3 years. We then calculated the percentage of patients with VL testing in 2018 and 2020 but not 2019, relative to those with testing in all 3 years.

#### Generalizability.

To assess whether Healthix HIV VL data are representative of NYC as a whole, we applied the NYC DOHMH's methodology for estimating citywide HIV viral suppression to Healthix data. The NYC DOHMH selects the last VL measurement from each patient with HIV in a given year to generate a snapshot of viral suppression, so we selected the last VL measurement from every person with HIV in the Healthix data set in a given year and compared our calculated rate of viral suppression with the estimate published by the DOHMH in its annual NYC HIV surveillance report to see how closely data aligned.

### Statistical Methods

Conventional descriptive data analyses were conducted. We assessed differences in proportions using chi-square tests and differences in continuous variables using independent *t* tests. We applied 2-sided hypothesis testing and treated *P* < .05 as statistically significant. We conducted unadjusted and adjusted logistic regressions to generate ORs with 95% CIs to estimate the association between cohort and viral suppression (Table 2), as well as to describe the modified association between race/ethnicity and viral suppression within each cohort (Table 3). We also generated adjusted ORs, which adjusted for gender and borough of residence. Patients with missing gender and/or borough data were excluded from the adjusted OR calculations.

**Table 2.** Viral Suppression (≤200 Copies/mL) Between Cohorts A and B

Viral Load	Suppressed	Total	% Suppressed	Odds Ratio (95% CI)
Last of 2019				
Cohort A^a^	10 345	11 646	88.8	1 [Reference]
Cohort B^b^	2703	3136	86.2	0.79 (.70–.88)
First of 2021				
Cohort A	10 320	11 645	88.6	1 [Reference]
Cohort B	2557	3138	81.5	0.57 (.51–.63)
Last of 2022				
Cohort A	9302	10 255	90.7	1 [Reference]
Cohort B	2016	2313	87.2	0.70 (.61–.80)

Odds ratios were generated with logistic regression. All data are drawn from Healthix, a large regional health information exchange based in New York City.

^a^People living with HIV who received HIV care in 2020.

^b^People living with HIV who did not receive HIV care in 2020.

**Table 3.** Viral Suppression Among People Living With HIV by Cohort and Race/Ethnicity

	Cohort A^a^				Cohort B^b^			
Viral Load: Race/Ethnicity	Suppressed	Total	OR (95% CI)	aOR (95% CI)^c^	Suppressed	Total	OR (95% CI)	aOR (95% CI)^c^
Last of 2019								
Black	2893	3341	0.49 (.40–.61)	0.52 (.42–.64)	843	1050	0.32 (.22–.46)	0.38 (.26–.56)
Hispanic	2302	2572	0.65 (.52–.81)	0.70 (.55–.87)	491	579	0.44 (.29–.65)	0.53 (.35–.81)
Other^d^	1558	1721	0.73 (.57–.93)	0.70 (.55–.90)	349	391	0.65 (.41–1.04)	0.67 (0.41–1.07)
White	1609	1732	1 [Reference]	1 [Reference]	459	495	1 [Reference]	1 [Reference]
First of 2021								
Black	2851	3342	0.46 (.37–.56)	0.50 (.40–.62)	771	1049	0.33 (.23–.44)	0.39 (.28–.54)
Hispanic	2289	2572	0.64 (.51–.79)	0.69 (.55–.87)	467	579	0.49 (.34–.69)	0.61 (.42–.88)
Other^d^	1577	1720	0.87 (.67–1.11)	0.85 (.66–1.10)	344	391	0.86 (.57–1.31)	0.97 (.63–1.49)
White	1603	1729	1 [Reference]	1 [Reference]	443	495	1 [Reference]	1 [Reference]
Last of 2022								
Black	2594	2934	0.53 (.42–.67)	0.58 (.45–.74)	674	827	0.39 (.25–.59)	0.45 (.28–.68)
Hispanic	2163	2354	0.79 (.61–1.01)	0.88 (.67–1.14)	405	453	0.76 (.46–1.22)	0.91 (.54–1.49)
Other^d^	1407	1514	0.92 (.69–1.22)	0.94 (.70–1.25)	247	272	0.88 (.51–1.56)	0.92 (.52–1.63)
White	1379	1475	1 [Reference]	1 [Reference]	324	353	1 [Reference]	1 [Reference]

All data are drawn from Healthix, a large regional health information exchange based in New York City.

Abbreviations: aOR, adjusted odds ratio; OR, odds ratio.

^a^People living with HIV who received HIV care in 2020.

^b^People living with HIV who did not receive HIV care in 2020.

^c^Adjusted for gender and borough of residence.

^d^Due to low cell frequencies, patients were classified as *other* if they were initially classified as Asian, Native American or Alaska Native, Native Hawaiian or Pacific Islander, other, and more than 1 race.

We performed several sensitivity analyses (supplementary material). In the first sensitivity analysis, PLWH were required to have ≥2 VL tests over ≥2 years but were not required to have an *ICD* or SNOMED code corresponding to HIV (Supplementary Sensitivity Analysis 1). In the second sensitivity analysis, we defined VLS as ≤50 copies/mL rather than ≤200 copies/mL and re-created Table 2 using this alternative definition (Supplementary Sensitivity Analysis 2).

All analyses were performed with the R programming environment (version 4.1.1; R Foundation for Statistical Computing).

## RESULTS

We identified 34 611 PLWH, of which 11 653 patients met criteria for cohort A and 3141 met criteria for cohort B. Men were more prevalent than women in cohort A (71% vs 27%) and cohort B (70% vs 29%; Table 1). There was no significant difference in gender distribution between the cohorts (*P* = .31). Black or African American patients made up a larger share of cohort B as compared with cohort A (33.4% vs 28.7%). The median age of both cohorts was 51 years based on available data in 2019 (98.9% of patients represented).

### VLS and Receipt of Care

In 2019, members of cohort B had a lower mean number of annual VL tests than members of cohort A (1.99 vs 2.23, *P* < .001). Also in 2019, 86.2% of patients in cohort B were virally suppressed, as compared with 88.8% of cohort A, representing 21% lower odds of viral suppression (OR, 0.79; 95% CI, .70–.88).

**Figure 3.**
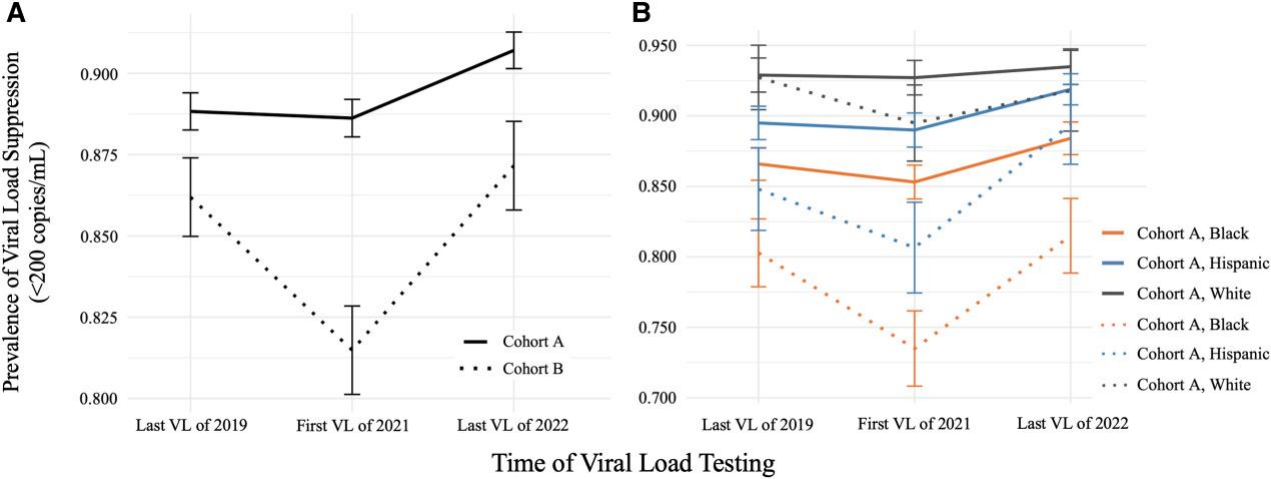
Prevalence of HIV viral load (VL) suppression (≤200 copies/mL) among 14 794 people living with HIV in the greater New York City region by cohort (*A*) and race/ethnicity and cohort (*B*). Error bars indicate 95% confidence intervals. All data are drawn from Healthix, a large regional health information exchange.

When members of cohort B returned to care in 2021, the prevalence of VLS had decreased significantly from 86.2% to 81.5% (*P* < .001), while it remained constant in cohort A over the same period (88.8% vs 88.6%, *P* = .62; Figure 3). Among patients with a VL measurement in 2022, 90.7% and 87.2% of cohorts A and B, respectively, had achieved VLS. However, we found that cohort B was less likely than cohort A to have had a VL test in 2022 (73.6% vs 88.0%, *P* < .001).

Geographically, the lowest prevalence of VLS was seen in the areas of Fordham–Bronx Park, High Bridge–Morrisania, and Hunts Point–Mott Haven of the Bronx among members of cohort B in 2021 (Figure 2).

### Racial and Ethnic Disparities in HIV Viral Suppression During the COVID-19 Pandemic

Racial and ethnic disparities in HIV viral control existed within both cohorts and across most time points. At baseline in 2019, Black patients were less likely than White patients to be virally suppressed in cohort A (OR, 0.49; 95% CI, .40–.61) and cohort B (OR, 0.32; 95% CI, .22–.46).

Between 2019 and 2021, racial and ethnic disparities persisted in both cohorts. These disparities were especially pronounced in cohort B in 2021, with 89.5% of White patients achieving VLS as compared with 73.4% of Black patients (OR, 0.33; 95% CI, .23–.44) and 80.7% of Hispanic patients (OR, 0.49; 95% CI, .34–.69). Racial and ethnic disparities also persisted in cohort A in 2021, with Black patients having lower odds of viral suppression than White patients (OR, 0.46; 95% CI, .37–.56). As of the last VL measurement of 2022, racial disparities had not resolved within either cohort.

Adjusting for gender and borough of residence did not change the significance of any of the aforementioned ORs (Table 3).

### Role of COVID-19

Comparing receipt of HIV care between 2019 and 2020 revealed that patients were significantly more likely to be lost to follow-up during 2020 vs 2019: 22.0% of PLWH were lost to follow-up in 2020 as opposed to 9.1% in 2019 (*P* < .001).

### Generalizability

Healthix estimates for VLS were within 1% point of DOHMH estimates in 3 of the 4 years assessed: 2018 (87% vs 87%), 2019 (88% vs 87%), 2020 (88% vs 86%), and 2021 (88% vs 87%) [18, 26–28].

Sensitivity analyses performed with an alternative definition of PLWH and an alternative cutoff for VLS yielded similar estimated odds of VLS by cohort and race/ethnicity as compared with the original definitions of PLWH and VLS (supplementary material).

## DISCUSSION

Using large-scale HIE data, we demonstrate that a larger proportion of PLWH were lost to follow-up in 2020 (the onset of the COVID-19 pandemic) as compared with surrounding years and that HIV viral suppression suffered in patients who did not receive HIV care in 2020. These disparities existed even before the COVID-19 pandemic: in 2019, members of cohort B had a lower prevalence of VLS and fewer annual VL tests than cohort A, indicating that viral control was already worse among the cohort that would go on to be lost to follow-up in 2020. The gap between cohorts A and B widened over the course of 2020 as the prevalence of viral suppression dropped in cohort B.

These findings support our hypothesis that PLWH who did not come in for VL testing during the early COVID-19 pandemic did not fare as well as those who continued VL monitoring. Moreover, it suggests that participants lacking VL tests in 2020 were not primarily PLWH with good medication adherence and sustained viral suppression who might have less urgent need to come in for VL testing during the early pandemic. Members of cohort B were also less likely to be currently in receipt of HIV care, suggesting that ongoing outreach efforts will be needed to reach those lost to care during 2020.

The COVID-19 pandemic highlighted racial and ethnic disparities across multiple stages of the HIV care continuum. Black patients made up a larger share of patients who did not undergo HIV VL testing in 2020 (cohort B) when compared with patients with HIV VL tests in the same year (cohort A), suggesting that they may have been more likely to fall out of HIV care than their White and Hispanic counterparts during the early days of the COVID-19 pandemic. This is consistent with past studies of racial disparities in VLS, which have shown greater barriers to care among Black PLWH [29, 30]. Furthermore, we found that the prevalence of viral suppression was lower among Black and Hispanic patients across cohorts. This indicates that racial and ethnic disparities persist even after controlling for receipt of HIV care. Geography also cannot completely account for these disparities, as adjusting for borough of residence did not significantly change any of the identified associations.

Overall, the lowest rates of viral control were among Black and Hispanic patients who did not receive HIV care, supporting the need for culturally informed care models and community-led interventions to improve health care access among members of these communities. Many approaches aimed at increasing retention in care have been suggested in the literature. In one recent qualitative study, PLWH who identified as Black and/or Latino noted several clinic-level factors as potential avenues for decreasing loss to follow-up and retention in care, including conveniently located multipurpose HIV care facilities, respectful provider-patient communication strategies, and individualized HIV treatment plans [31]. It is likely that a combination of these and other commonly cited tools, along with telehealth interventions and the diversification of the health care team, will be necessary to fully bridge the HIV care gap [32].

Our work suggests that HIEs present HIV researchers with the opportunity to combine the scope of health department data with the granularity of local EHR data. In NYC, the gold standard for HIV-related reporting is the NYC DOHMH, which publishes annual estimates of HIV viral suppression among individuals in receipt of HIV care. Using HIE data, we generated annual viral suppression estimates that were within 1 percentage point of DOHMH estimates in 3 of the 4 years that we evaluated. This strong concordance between our viral suppression estimates and the DOHMH estimates supports our assumption that Healthix data are representative of PLWH in NYC. The generalizability of Healthix data will likely only increase as more facilities are added. While this analysis did not include the medication and health care encounter data that HIEs often contain, such data can be used explore medication adherence, comorbidities, and individual-level socioeconomic determinants of health.

### Limitations

This analysis has several limitations that should be considered. While reasonably comprehensive in its coverage, Healthix does not receive data from all facilities in the greater NYC area. Due to the lack of a comprehensive list of facilities that do not participate in Healthix, we were unable to determine if estimates were biased by over- or underrepresentation of certain clinic types or locations. While we cannot account for this, the fact that our overall data closely match NYC DOHMH estimates is reassuring. Furthermore, as not all facilities were included, we cannot exclude the possibility that some patients received additional VL testing at other facilities. While it is possible that patients moved and/or had VL testing at other facilities during the pandemic, requiring that patients in cohorts A and B have a VL test in 2021 gives us confidence that they did not move out of the region permanently or pass away during 2020.

A significant limitation is the issue of missing race and ethnicity data. Approximately 20% of patients in both cohorts were missing race data, and an additional 11% to 13% were classified as *other*. Unfortunately, this issue is not unique to Healthix or even HIEs but common with the use of EHR data, leading to calls for improved racial and ethnic data collection at medical centers [33, 34]. It is difficult to know in which direction, if any, these missing data may bias our analyses that pertain to racial and ethnic disparities. Certainly, our decision to exclude individuals with missing race data from risk calculations involving race reduces the power of those analyses.

Another limitation to this analysis is the lack of a standardized definition of PLWH in the context of HIE data. Diagnostic codes are not entirely reliable, as providers may inadvertently use HIV diagnosis codes instead of HIV testing codes and vice versa. Similarly, HIV VL tests may be inappropriately ordered as screening tests, so the receipt of a single HIV VL test does not necessarily indicate true HIV infection. However, the sensitivity analysis that we performed using an alternative definition of PLWH yielded similar results when compared with our original analysis (supplementary material, Supplementary Sensitivity Analysis 1). This allows us to be somewhat confident that our conclusions are not contingent upon which definition of PLWH that we apply.

In this study, 2020 is used as a proxy for the height of COVID-19–related societal disruptions, as this was the year that NYC was most affected by COVID-19 deaths, lockdowns, and business closures. It is possible that other temporal factors could have affected receipt of care, but given the size and breadth of the HIE, we are not aware of any other care disruptions of this magnitude in 2020.

## CONCLUSIONS

In the NYC region, HIV viral suppression dropped among PLWH who did not receive HIV care in 2020. This burden was borne disproportionately by Black and Hispanic patients, who are overrepresented in the HIV epidemic, underscoring the necessity for policy makers to target resources to these communities with greatest need during public health crises. These policies must be driven by timely data, which HIEs offer. Health departments typically release HIV data annually or semiannually, while HIEs offer the opportunity to guide policy decisions in near real time. Using data from HIEs and health departments would allow researchers to identify concerning trends sooner and adapt policies to meet the needs of rapidly evolving situations, such as COVID-19.

## Supplementary Material

ofad584_Supplementary_Data

## Supplementary Data

Supplementary materials are available at *Open Forum Infectious Diseases* online. Consisting of data provided by the authors to benefit the reader, the posted materials are not copyedited and are the sole responsibility of the authors, so questions or comments should be addressed to the corresponding author.

## Notes

***Acknowledgments.*** The authors thank Healthix for providing the data necessary to perform study analyses.

***Author contributions.*** All authors had full access to the data in the study. They also take responsibility for the integrity of the data and accuracy of the analysis and have approved the final manuscript. Concept and design: all authors. Acquisition, analysis, or interpretation of data: all authors. Drafting of the manuscript: all authors. Critical revision of the manuscript for important intellectual content: all authors. Statistical analysis: all authors. Obtained funding: all authors. Administrative, technical, or material support: all authors. Supervision: all authors.

***Data availability.*** The EHR and administrative data underlying this article are not available due to restrictions to preserve patient confidentiality.

***Patient consent statement.*** This study was approved by the institutional review board of the Columbia University Irving Medical Center (protocol AAAT1774) with a waiver of informed consent.

***Financial support*.** This work was supported by grants from the National Institute of Allergy and Infectious Diseases and the National Library of Medicine at the National Institutes of Health (T15-LM007079 to H. R. N.; K23-AI150378 and UM1AI069470 to J. Z.); and by the 2022 Infectious Disease Society of America's Grant for Emerging Researcher/Clinician Mentorship Program (to E. T.).
