# Supplementary material for: Using a Health Information Exchange to Characterize Changes in HIV Viral Load Suppression and Disparities During the COVID-19 Pandemic in New York City
Source: Open Forum Infect Dis. 2023 Nov 29;10(12):ofad584. doi: 10.1093/ofid/ofad584 (PMC10754646; doi:10.1093/ofid/ofad584)
Supplement: ofad584_Supplementary_Data [file ofad584_supplementary_data.docx]

**SUPPLEMENTAL MATERIALS**

**Sensitivity analysis 1**

We performed sensitivity analyses to test the effect of using a different definition of PLWH. Our original definition of PLWH defines PLWH as anyone with ≥2 HIV VL tests over ≥2 years as well as an ICD or SNOMED code corresponding to HIV. Our alternative definition defines PLWH as anyone with ≥2 VL tests over ≥2 years but does not require an ICD or SNOMED code corresponding to HIV.

Using this alternative definition, we identified 40,985 PLWH (vs 34,611 originally). The demographic makeup of PLWH using the original and alternative definitions is shown in S1 Table 1. The two groups of PLWH differed by racial/ethnic identity and borough of residence, though not by gender.

***S1 Table 1.* Baseline demographic characteristics** of all PLWH using the original definition of PLWH (≥2 HIV VL tests over ≥2 years plus an ICD or SNOMED code corresponding to HIV) vs an alternative definition of PLWH (≥2 VL tests over ≥2 years +/- an ICD or SNOMED code corresponding to HIV). All data is drawn from Healthix, a large regional health information exchange.

|  | **PLWH (original definition)** | | **PLWH (alternative definition)** | | **X^2^ p value** |
| --- | --- | --- | --- | --- | --- |
|  | **n** | **% (95% CI)** | **n** | **% (95% CI)** |  |
| **Overall** | 34611 |  | 40985 |  | 0.15 |
| **Gender** |  |  |  |  |  |
| Female | 9345 | 27.0 (26.5-27.5) | 10827 | 26.4 (26.0-26.8) |  |
| Male | 24877 | 71.9 (71.4-72.3) | 29716 | 72.5 (72.1-72.9) |  |
| Other, unknown, or declined | 389 | 1.1 (1.0-1.2) | 442 | 1.1 (1.0-1.2) |  |
| **Race** |  |  |  |  | < 0.001 |
| Asian | 776 | 2.2 (2.1-2.4) | 991 | 2.4 (2.3-2.6) |  |
| Black or African American | 11223 | 32.4 (31.9-32.9) | 12559 | 30.6 (30.2-31.1) |  |
| Hispanic or Latino | 7898 | 22.8 (22.4-23.3) | 9048 | 22.1 (21.7-22.5) |  |
| Native American or Alaska  Native | 116 | 0.3 (0.3-0.4) | 138 | 0.3 (0.3-0.4) |  |
| Native Hawaiian or Pacific  Islander | 49 | 0.1 (0.1-0.2) | 60 | 0.1 (0.1-0.2) |  |
| White | 5524 | 16.0 (15.6-16.3) | 6844 | 16.7 (16.3-17.1) |  |
| More than one race | 22 | 0.1 (0.0-0.1) | 25 | 0.1 (0.0-0.1) |  |
| Other race | 3569 | 10.3 (10.0-10.6) | 4345 | 10.6 (10.3-10.9) |  |
| Unknown/Declined | 5434 | 15.7 (15.3-16.1) | 6975 | 17.0 (16.7-17.4) |  |
| **Borough of residence** | | | | | 0.01 |
| Bronx | 8265 | 23.9 (23.4-24.3) | 9928 | 24.2 (23.8-24.6) |  |
| Brooklyn | 7146 | 20.6 (20.2-21.1) | 8309 | 20.3 (19.9-20.7) |  |
| Manhattan | 8677 | 25.1 (24.6-25.5) | 10308 | 25.2 (24.7-25.6) |  |
| Queens | 5415 | 15.6 (15.3-16.0) | 6217 | 15.2 (14.8-15.5) |  |
| Staten Island | 934 | 2.7 (2.5-2.9) | 1009 | 2.5 (2.3-2.6) |  |
| Non-NYC | 3960 | 11.4 (11.1-11.8) | 4932 | 12.0 (11.7-12.3) |  |
| Unknown | 214 | 0.6 (0.5-0.7) | 282 | 0.7 (0.6-0.8) |  |

Among the 40,985 PLWH identified using the alternative definition, 12,958 met criteria for Cohort A (received HIV care in 2020) and 3,362 met criteria for Cohort B (did not receive HIV care in 2020). Using these alternative cohorts, we recreated the logistic regression models used in the original analysis to quantify the association between cohort and odds of viral suppression (**S1 Table 2a**), as well as to describe the association between race/ethnicity and viral suppression within each cohort (**S1 Table 2b**). We also generated adjusted odds ratios, which adjusted for gender and borough of residence (**S1 Table 2b**).

***S1 Table 2a.*** Viral suppression among PLWH in NYC by cohort, using the original definition of PLWH (≥2 VL tests over ≥2 years plus an ICD or SNOMED code corresponding to HIV) and an alternative definition of PLWH (≥2 VL tests over ≥2 years +/- an ICD or SNOMED code corresponding to HIV). Odds ratios generated using logistic regression. All data is drawn from Healthix, a large regional health information exchange.

|  |  | **PLWH (original definition)** | | **PLWH (alternative definition)** | |
| --- | --- | --- | --- | --- | --- |
|  |  | **% Suppressed** | **OR (95% CI)** | **% Suppressed** | **OR (95% CI)** |
| **Last VL of 2019** | Cohort A | 88.8 | Reference | 89.0 | Reference |
|  | Cohort B | 86.2 | 0.79 (0.70-0.88) | 86.6 | 0.80 (0.71-0.90) |
| **First VL of 2021** | Cohort A | 88.6 | Reference | 88.8 | Reference |
|  | Cohort B | 81.5 | 0.57 (0.51-0.63) | 82.0 | 0.58 (0.52-0.64) |
| **Last VL of 2022** | Cohort A | 90.7 | Reference | 90.9 | Reference |
|  | Cohort B | 87.2 | 0.70 (0.61-0.80) | 87.5 | 0.70 (0.62-0.81) |

***S1 Table 2b.*** Viral suppression among PLWH in NYC by cohort and race/ethnicity, using an alternative definition of PLWH (≥2 VL tests over ≥2 years +/- an ICD or SNOMED code corresponding to HIV). Odds ratios generated using logistic regression. All data is drawn from Healthix, a large regional health information exchange.

|  | | |  | **Cohort A (alternative definition)** | | | | **Cohort B (alternative definition)** | | | | | |
| --- | --- | --- | --- | --- | --- | --- | --- | --- | --- | --- | --- | --- | --- |
|  | | |  | % suppressed | | OR (95% CI) | aOR (95% CI) † | % suppressed | | | OR (95% CI) | | aOR (95% CI) † |
| **Last VL of 2019** | | | Black | 86.7 | | 0.46 (0.37-0.56) | 0.48 (0.39-0.59) | 81.0 | | | 0.34 (0.24-0.49) | | 0.42 (0.29-0.60) |
|  |  |  | Hispanic | 89.4 | | 0.59 (0.47-0.73) | 0.63 (0.50-0.79) | 85.4 | | | 0.48 (0.32-0.70) | | 0.59 (0.39-0.88) |
|  |  |  | Other* | 90.5 | | 0.67 (0.53-0.84) | 0.65 (0.51-0.82) | 89.7 | | | 0.70 (0.45-1.09) | | 0.73 (0.47-1.15) |
|  |  |  | White | 93.4 | | Reference | Reference | 92.5 | | | Reference | | Reference |
|  |  |  | | |  |  |  |  |  |  | |  |  |
| **First VL of 2021** | | | Black | 85.4 | | 0.44 (0.36-0.53) | 0.48 (0.39-0.59) | 74.2 | | | 0.33 (0.24-0.45) | | 0.41 (0.29-0.56) |
|  |  |  | Hispanic | 89.0 | | 0.61 (0.49-0.75) | 0.66 (0.53-0.83) | 81.4 | | | 0.51 (0.36-0.71) | | 0.65 (0.45-0.93) |
|  |  |  | Other* | 87.1 | | 0.82-0.65-1.03) | 0.81 (0.64-1.03) | 88.0 | | | 0.86 (0.57-1.28) | | 0.98 (0.65-1.48) |
|  |  |  | White | 93.0 | | Reference | Reference | 89.6 | | | Reference | | Reference |
|  |  |  | | |  |  |  |  |  |  | |  |  |
| **Last VL of 2022** | | | Black | 88.4 | | 0.52 (0.41-0.64) | 0.56 (0.44-0.70) | 82.1 | | | 0.40 (0.26-0.60) | | 0.46 (0.30-0.70) |
|  |  |  | Hispanic | 91.9 | | 0.76 (0.60-0.97) | 0.84 (0.65-1.08) | 89.7 | | | 0.77 (0.47-1.22) | | 0.93 (0.57-1.52) |
|  |  |  | Other* | 93.0 | | 0.89 (0.68-1.16) | 0.91 (0.70-1.20) | 90.8 | | | 0.86 (0.50-1.47) | | 0.90 (0.53-1.56) |
|  |  |  | White | 93.7 | | Reference | Reference | 91.9 | | | Reference | | Reference |

*Patients were classified as “Other” if they were initially classified as “Asian,” “Native American or Alaska Native,” “Native Hawaiian or Pacific Islander,” “Other,” and “More than one race,” due to low cell frequencies.

† Adjusted for gender and borough of residence

*Abbreviations:* PLWH, persons living with HIV. NYC, New York City. VL, viral load.

Use of the alternative definition of PLWH did not change the significance of any of the originally identified associations between cohort and odds of VLS (**S1 Table 2a**). Use of the alternative definition of PLWH also did not change the significance of the originally identified associations between race/ethnicity and odds of VLS within each cohort, with one exception: the unadjusted odds ratio comparing VLS among Hispanic and white PLWH in Cohort A in 2022 was originally nonsignificant (95% CI 0.61-1.02) but became significant when the alternative definition was applied (95% CI 0.61-0.99) (**Table 2b, S1 Table 2b**).

The strong concordance between the odds ratios generated using the original definition of PLWH and those generated using the alternative definition of PLWH gives us confidence that our findings are robust and not contingent upon whether or not we require HIV diagnostic codes in addition to multiple HIV viral loads to meet our definition of PLWH.

**Sensitivity Analysis 2**

We performed an additional sensitivity analysis in which we used an alternative definition of HIV VLS (**≤**50 copies/mL) and recreated Table 2a using this alternative definition.

Our original definition defined VLS as **≤**200 copies/mL, consistent with the DOHMH definition of VLS. As expected, we found a lower prevalence of viral suppression among both cohorts using the alternative definition. However, this alternative definition did not change the significance of any of the comparisons between Cohorts A and B (Table 2a, S3 Table 2a).

***S2 Table 2a.*** Viral suppression (**≤** 50 copies/mL) among Cohort A (PLWH who received care in 2020) and Cohort B (PLWH who did not receive care in 2020). Odds ratios generated using logistic regression. All data is drawn from Healthix, a large regional health information exchange.

|  |  | **Suppressed** | **Total** | **% Suppressed** | **OR (95% CI)** |
| --- | --- | --- | --- | --- | --- |
| **Last VL of 2019** | Cohort A | 9406 | 11623 | 80.9 | Reference |
|  | Cohort B | 2458 | 3132 | 78.5 | 0.86 (0.78-0.95) |
| **First VL of 2021** | Cohort A | 9451 | 11638 | 81.2 | Reference |
|  | Cohort B | 2331 | 3138 | 74.3 | 0.67 (0.61-0.73) |
| **Last VL of 2022** | Cohort A | 8449 | 10233 | 82.6 | Reference |
|  | Cohort B | 1818 | 2310 | 78.7 | 0.78 (0.70-0.87) |

The consistency between the odds ratios generated using the original definition of VLS (**≤**200 copies/mL) and those generated using the alternative definition of VLS (**≤**50 copies/mL) gives us further confidence that the significance of our findings is not contingent upon a specific VLS cutoff.
